# Exploration of the Polymorphism Distribution of Bovine *HMGA2* Gene in Worldwide Breeds and Its Associations with Ovarian Traits

**DOI:** 10.3390/ani14050796

**Published:** 2024-03-04

**Authors:** Siyuan Shen, Leijing Zhu, Yuanzhe Yang, Yi Bi, Jie Li, Yongsheng Wang, Chuanying Pan, Shuilian Wang, Xianyong Lan

**Affiliations:** 1 College of Animal Science and Technology, Northwest A&F University, Yangling 712100, China; ssy991202@163.com (S.S.); zhuleijingjingshu@163.com (L.Z.); yangyuanzhe9@163.com (Y.Y.); biyi0312@163.com (Y.B.); lijie950302@163.com (J.L.); panyu1980@126.com (C.P.); 2 College of Veterinary Medicine, Northwest A&F University, Yangling 712100, China; wangyongsheng01@nwsuaf.edu.cn; 3 College of Veterinary Medicine, Hunan Agricultural University, Changsha 410125, China

**Keywords:** bovine, *HMGA2*, insertion–deletion (indel), fertility, ovarian traits

## Abstract

**Simple Summary:**

Bovine breeding is an economically important endeavor in China. Ovarian traits play a significant role in the reproductive characteristics. This study aimed to underscore the discovery of a novel indel locus within the *HMGA2* gene in Holstein cows. Our study found that a variant in the bovine *HMGA2* gene has a significant connection with ovarian traits. These findings contribute to a more promising outlook for the bovine industry.

**Abstract:**

The high-mobility group AT-hook 2(*HMGA2*) gene has been widely studied in the context of cancer and animal growth. However, recently, several studies have uncovered its critical role in cell proliferation. A genome-wide association study (GWAS) further suggests that the *HMGA2* gene is a candidate gene in fertility, indicating its connection not only to growth traits but also to reproduction, specifically ovarian traits. Thus, this study aimed to analyze the distribution of the *HMGA2* gene in 54 bovine breeds worldwide, identify important short fragment variants (indels), and investigate the relationship between *HMGA2* and ovarian development. The dataset included genotypic information from a bovine population of 634 individuals (n = 634). After genotyping and analyzing four selected loci, we found that one out of four loci, rs133750033 (P4-D_22-bp_), was polymorphic. Our results also reveal that this indel of *HMGA2* is significantly associated with certain ovarian traits (*p <* 0.05). Specifically, it has connection with ovarian length (*p* = 0.004) and ovarian height (*p* = 0.026) during diestrus. Additionally, we discovered a higher expression of the *HMGA2* gene in Asian cattle breeds. In summary, this study suggests that *HMGA2* has the potential to serve as an animal fertility testing marker gene. Moreover, these findings contribute to a more promising outlook for the bovine industry.

## 1. Introduction

Boosting the reproductive capacity of female livestock has been an ongoing economic priority in animal breeding. Specifically in the cattle sector, the persistent selection of high-producing dairy cows has resulted in a decline in female fertility [1]. Consequently, in recent times, there has been a growing focus on research aimed at enhancing bovine fertility. Various metrics, such as the development of ovaries and the caliber of mature ovarian follicles, are employed to assess cattle’s reproductive capabilities. The optimal development of reproductive organs, including ovaries and testes, is associated with the production of superior quality gametes (sperm and ova). This is particularly vital in bovines, where the quality of mature follicles is crucial for the efficacy of artificial insemination techniques. Following the release of follicles by the ovaries, structures akin to ovarian glands, notably the corpus luteum, undergo rapid transformation from these follicles. The corpus luteum functions as a ‘temporary gland’, primarily engaged in the synthesis and release of progesterone. It may also undergo transformation or decay into the white body, playing a critical role in altering or sustaining the receptivity of the endometrium for pregnancy. Thus, identifying key genes influencing the functions of the ovaries, corpus luteum, and related characteristics is fundamental for employing molecular marker-assisted selection techniques to augment cattle fertility [2,3].

Fertility in cattle is a multifaceted characteristic influenced by numerous genes and mutations that are involved in reproductive processes, spanning a range of biological pathways. In the recent era, fueled by the swift advancements in sequencing technologies, there is an emerging trend to elucidate the underlying causes and mechanisms of complex traits in Holstein cows. This is achieved through comprehensive genome sequencing and bioinformatics analyses, encompassing the estimation of genetic substitution rates, exploring associations between milk composition and breeding values, and identifying potential candidate genes linked to longevity, fertility, and other intricate traits [4]. Bovine breeding is also an important economic issue in China. Therefore, the study of how to improve the breeding capacity of bovine remains a top priority. The ovary, a crucial organ in female reproduction, is responsible for producing ova and secreting estrogen. Numerous studies have highlighted that the morphology of the ovary, influenced by various elements such as hormonal levels and genetic factors, directly correlates with a female’s reproductive capabilities [5]. It has been consistently observed that young, non-pregnant cows with larger ovaries exhibit a greater quantity of follicles and oocytes. Thus, the structure and functionality of the ovary play pivotal roles in the regulation of reproductive processes [6].

The high-mobility group AT-hook 2 (*HMGA2*) gene is well known for its strong connection with animal growth [7]. First of all, it was found that the gene is significantly associated with human height [8]. Studies also indicate a strong association between the *HMGA2* gene and cancer. For example, *HMGA2* is dysregulated in a wide array of human malignancies, encompassing lung, breast, and ovarian cancers, where its heightened expression is linked to an increased likelihood of cancer advancement [9]. RNA sequencing comparisons between cancerous and normal tissues have revealed diverse expression patterns of *HMGA2* across various cancer types [10]. 

Typically, *HMGA2* shows greater expression in sarcomas, brain and central nervous system tumors, as well as in cancers of the esophagus, head and neck, lung, melanoma, ovary, and pancreas, relative to their normal tissue counterparts. Subsequently, researchers observed that the influence of this gene extended to animals, establishing notable associations, such as its association with the body sizes of horses and dogs [10,11,12]. The *HMGA2* gene with respect to growth regulation is highly conserved among mammals [13]. Research indicates a potential association between *HMGA2* and ovarian traits [14]. Recent investigations in oncology have illuminated the significant involvement of the *HMGA2* in key processes of tumor formation, such as the epithelial–mesenchymal transition (EMT) and the development of new blood vessels (angiogenesis). Studies suggest that *HMGA2* plays an essential role in controlling angiogenesis in both endothelial cells and their progenitor counterparts. Notably, a study focusing on oral squamous cell carcinoma revealed a noteworthy upregulation in *HMGA2* accompanied by a heightened expression in angiogenic genes such as vascular endothelial growth factor (VEGF)-A, VEGF-C, and fibroblast growth factor-2 in these tumors. This upregulation contributes to a marked increase in blood vessel formation, emphasizing the pivotal role of the *HMGA2* gene in angiogenesis regulation. This link between *HMGA2* overexpression and elevated vascular density may potentially contribute to tumor formation [15]. The overexpression of *HMGA2* is observed in a variety of mesenchymal tumors across multiple organ systems, including those in the head and neck, lungs, bones, breast, and female reproductive organs. Notably, this overexpression is a common feature in uterine smooth muscle tumors and other mesenchymal neoplasms of gynecological origin, where specific molecular and genetic alterations result in the increased expression of *HMGA2* [16]. Most recently, as a candidate risk gene, *HMGA2* was suggested to be related to polycystic ovary syndrome (PCOS) [17]. Along with this, a new GWAS study has uncovered the fact that the *HMGA2* gene may also be a candidate gene for bovine fertility [18]. However, whether there is a connection between the *HMGA2* gene and bovine fertility traits remains obscure. 

Polycystic ovary syndrome (PCOS) represents the most prevalent endocrine disorder affecting the reproductive health of women in their childbearing years. It is characterized by hyperandrogenism and polycystic ovarian changes, often accompanied by insulin resistance (IR) and a series of metabolic abnormalities similar to the clinical manifestations of type 2 diabetes [19]. A genome wide association study (GWAS) revealed that high-mobility group A2 (*HMGA2*) is a common susceptibility gene for PCOS and type 2 diabetes [17]. So, we speculate that the *HMGA2* gene may also be associated with PCOS in bovine. Moreover, *HMGA2* is a non-histone chromosomal protein, with no transcriptional activity, and can regulate the transcription of other genes by altering chromosome structure.

In this study, we explored the potential role of *HMGA2* in bovine fertility by analyzing data from 634 cattle at various estrus stages. Our goal was to enhance the understanding of *HMGA2* and contribute to the ongoing development of cattle reproductive capabilities.

## 2. Materials and Methods

### 2.1. Bovine Ovary Collection

We assembled a comprehensive set of 634 ovary specimens from healthy Holstein cows, all within the uniform age range of 5–6 years and raised under consistent environmental conditions. The samples were collected from fixed dairy farms in Shaanxi Province, and the feeding management and nutritional conditions were the same. The dimensions of each ovary sample, including height, width, length, and weight, were accurately measured by the same individual using a consistent methodology. Furthermore, we carefully documented the number of formations such as of the corpus luteum and corpus albicans, as well as mature follicles, and measured their individual diameters. The measurement tools included a sanitized electronic scale for ovary weight evaluation, and either a vernier caliper or a two-sided ruler to measure the height, width, and length of each ovary. Furthermore, we determined the estrus stage indirectly by typing the corpus luteum: a type 1 and type 2 corpus luteum indicated the late stages of estrus; types 3 and 4 suggested the dioestrum; the absence of follicles and corpus luteum on the ovaries indicated pre-estrus; while no corpus luteum and large follicles indicated estrus (corpus luteum type 1: conical; corpus luteum type 2: volcanic crater; corpus luteum type 3: mushroom shaped; corpus luteum type 4: flattened; corpus luteum type 5: no corpus luteum) [20]. For association analysis, we collected these portions of ovarian samples. The experiments of this study were approved by the International Animal Care and Use Committee of the Northwest A&F University (protocol number: NWAFAC1008).

### 2.2. InDel Loci Selection and Primer Design

The extraction of bovine genomic DNA was performed using the salt-extraction technique as detailed by Aljanabi and Martinez [21]. For an efficient PCR analysis, the DNA isolated from ovarian tissues was diluted to a concentration of 10 ng/µL. The purity of these samples was ascertained using a NanoDrop 1000 (Thermo Scientific, Waltham, MA, USA).

Utilizing the bovine *HMGA2* sequence available on NCBI (https://www.ncbi.nlm.nih.gov, accessed on 3 February 2022), we conducted a search in the Ensemble database (https://asia.ensembl.org/index.html, accessed on 4 February 2022) to identify indels longer than 10 bp, which are typically easier to genotype. This search led to the discovery of 2 indel loci in the 5′ UTR region (rs516271779, rs797520182) and another 2 in an intron (rs797609300, rs133750033), all associated with the bovine *HMGA2* gene (GeneID:100297175, NC_037332). A primer for each of these loci was designed using the NCBI’s primer design tool (https://www.ncbi.nlm.nih.gov/tools/primer-blast/, accessed on 4 February 2022) and subsequently synthesized by TSINGKE Biological Technology in Beijing, China, resulting in the development and preparation of 4 primers.

### 2.3. PCR Amplification and Genotyping

In our genotyping study, we employed 4 distinct primers to analyze polymorphisms across 4 indel loci (Table 1). A composite DNA sample, derived from 24 randomly chosen DNA extracts, exhibited polymorphic variations under a touch-down PCR method. The resulting PCR products, separated on 2.5% agarose gels, were individually amplified for further analysis. Following the Polymerase Chain Reaction (PCR) procedure conducted on a sample of 100 individuals, we analyzed the data for each locus and its association with ovarian features to explore the possible connections between locus polymorphisms and ovarian traits. The entire process and reaction setups adhered to the methodology outlined by Akhatayeva [22].

### 2.4. Statistical Analysis

The assessments of the polymorphism information content (PIC) and Hardy–Weinberg equilibrium (HWE) were executed using the GDIcall Online Calculator, adhering to the methodologies proposed by Nei [23]. 

To elucidate the relationship between genotypes and ovarian characteristics, we utilized a general linear model, defined as Yij = μ + Gi + Eij. Here, Yij represents the specific ovarian trait of the ijth cow, μ is the aggregate mean, Gi denotes the genotype of the ijth cow, and Eij corresponds to the random error component [24].

To examine the link between indel loci and ovarian characteristics, we employed both one-way ANOVA and the independent sample *t*-test, utilizing SPSS software version 23.0 (IBM Corp., Armonk, NY, USA). Our methodology involved analyzing indels that displayed 3 genotypes (II, ID, and DD), each with over 3 occurrences, through one-way ANOVA. For scenarios where any of these 3 genotypes presented less than 3 instances, we conducted an independent sample *t*-test. This extensive analysis with SPSS yielded clear findings on the possible association between indel loci and ovarian features. We considered results with a *p*-value below 0.05 to be statistically significant.

### 2.5. Worldwide Bovine Breeds’ Sample Information

Our data were collected from the database of BGVD (http://animal.omics.pro/code/index.php/BosVar, accessed on 3 July 2022), which contains 54 bovine breeds and 432 samples. The set of samples contains the following numbers of individuals: 108 West European, 28 Northeast Asian, 9 Middle East, 9 Tibetan, 47 North-Central Chinese, 21 Northwest Chinese, 33 South Chinese, 24 Indo-Pakistan, 83 Central-South European, and 70 African cattle.

This database also provides the signatures of a selection for 8 groups, 6 of which were the “core” cattle groups and were identified as ancestral groups (Indian indicine (IN), Chinese indicine (CN), East Asian taurine (EA), Eurasian taurine (EUA), European taurine (EUR), African taurine (AFR)). In this research, data processing and integration into the BGVD followed a methodology consistent with established protocols. Initially, Illumina short, 250 bp paired-end reads were mapped to the Btau 5.0.1 genome version (GCF_000003205.7) using the Burrows–Wheeler Aligner (BWA) algorithm, achieving an approximate 13× coverage across the bovine genome in various cattle breeds. Redundant reads were eliminated employing Picard tools (accessible at http://broadinstitute.github.io/picard/, accessed on 3 January 2019). Subsequently, the Genome Analysis Toolkit (GATK) facilitated the identification of SNPs and indels, uncovering roughly 60.4 million autosomal SNPs and about 6.8 million autosomal indels. The phasing of these SNPs was conducted using Beagle software (Version: 5.1). SnpEff was employed for the annotation of SNPs and indels. In utilizing PLINK, the minor allele frequency (MAF) was calculated for the entire cattle population, alongside allele frequencies for individual breeds and a defined “core” cattle group. CNVcaller software (Version: 0.1.1) was instrumental in detecting CNVs, resulting in the identification of 76,634 CNV regions (CNVR) across 432 cattle genomes, which were then annotated using Annovar. Considering the prevalent use of 3 bovine genome versions—Btau 5.0.1, UMD3.1.1, and the more recent ARS-UCD1.2 (project accession: NKLS00000000)—the conversion of variation coordinates to the formats of the latter 2 genomes was achieved through the creation of liftOver chain files (Btau5.0.1ToUMD3.1.1.chain.gz and Btau5.0.1ToARS-UCD1.2.chain.gz) and their subsequent application via liftOver [25]. The geographic information and other detailed information for each sample is provided on the homepage (http://animal.omics.pro/code/index.php/BosVar, accessed on 5 January 2019) and the corresponding “Sample Table” page.

Here, we analyzed the distribution of P4- rs133750033 in the *HMGA2* gene in 54 bovine breeds and 6 core bovine breeds.

## 3. Results

### 3.1. Identification of the Indels in Bovine HMGA2 Gene

**Figure 1:**
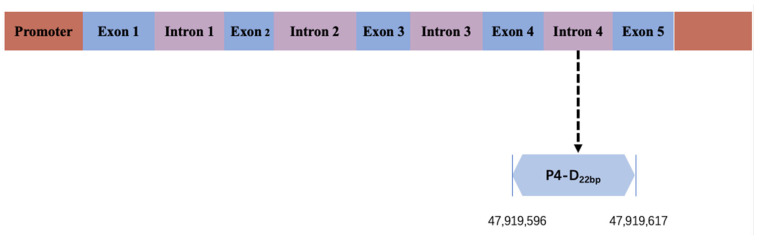
Gene structure of bovine *HMGA2* gene.

**Figure 2:**
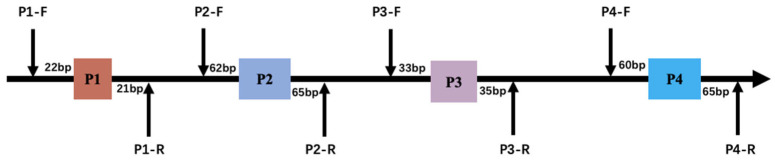
Primer diagram of bovine *HMGA2* gene.

**Figure 3:**
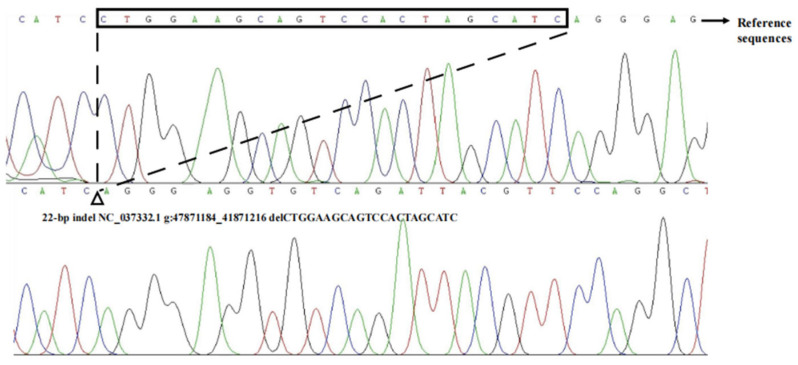
Sequence diagram of P4-D_22bp_ of bovine *HMGA2* gene.

**Figure 4:**
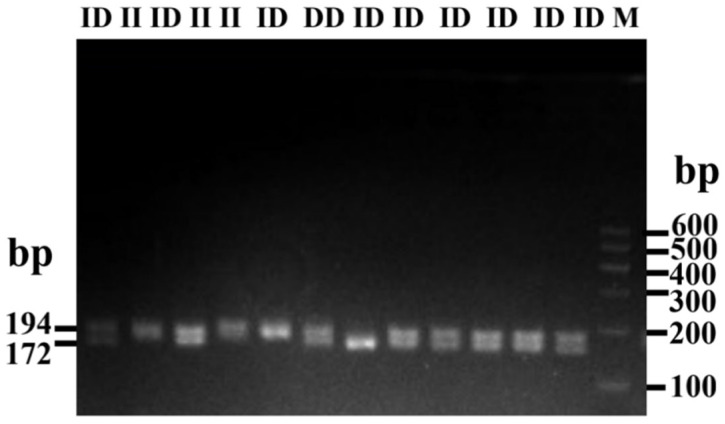
Electrophoresis pattern of P4-D_22bp_ of bovine *HMGA2* gene.

In the process of identifying genotypes within four indel loci using a pooled DNA approach, it was determined that two loci, P1-rs516271779 and P4-rs133750033, exhibited polymorphism, each displaying three genotype forms: homozygous insertion (II), homozygous deletion (DD), and heterozygous (ID) types (Figure S1, Table 1 and Table S1). The P4 locus was specifically located in the fourth intron of the *HMGA2* gene (Figure 1). The primer diagram of the bovine *HMGA2* gene is shown in Figure 2. The deletion sequence of P4, as revealed through direct sequencing, matched the sequence registered in the NCBI database (P4-22bp, CTGGAAGCAGTCCACTAGCATC) (Figure 3). Furthermore, the PCR products of P4, following a separation through 2.5% agarose gel electrophoresis, are displayed in Figure 4.

### 3.2. Estimation of Polymorphism Parameters of Indel (P4-D22-bp) of HMGA2

Utilizing the genetic variations found in the *HMGA2*-P4 sites, we computed the frequencies of genotypes and alleles, along with other population genetics parameters. Within the *HMGA2*-P4 locus, the occurrence of the DD genotype (0.48) surpassed that of the ID genotype (0.34) (Table 2).

To gain a more thorough understanding of the genotype distribution in the P1 and P4 regions, we estimated a range of polymorphism parameters. These encompassed the frequencies of genotypes and alleles, the Hardy–Weinberg equilibrium (HWE), and metrics such as homozygosity (Ho), heterozygosity (He), effective number of alleles (Ne), and polymorphism information content (PIC). For P1, the estimates were as follows: Ho: 0.52, He: 0.48, Ne: 1.84, and PIC: 0.35. Correspondingly, in P4, the estimates were Ho: 0.75, He: 0.25, Ne: 1.33, and PIC: 0.22.

### 3.3. Association Analysis of HMGA2 with Ovarian Traits

To determine the association of P1 and P4 with ovarian characteristics, we conducted an analysis of their relevance using SPSS 23. As previously mentioned, following the completion of PCR procedures in 100 individuals, we analyzed the relationship between each polymorphic locus and ovarian traits. Upon finalizing the PCR process for groups of 100, 200, 300, and 400 individuals, our findings consistently suggested a linkage between P4 and ovarian traits during diestrus. However, no significant association was observed between P1 and ovarian traits.

P4 was correlated with ovarian length (*p* = 0.004) and ovarian height (*p* = 0.026) during diestrus. Individuals with the II and ID genotypes possessed ovaries with greater length and height than those with the DD genotype (Table 3). 

### 3.4. Analysis of HMGA2 Gene Distribution in 54 Bovine Breeds and 6 Core Bovine Breeds Worldwide

**Figure 5:**
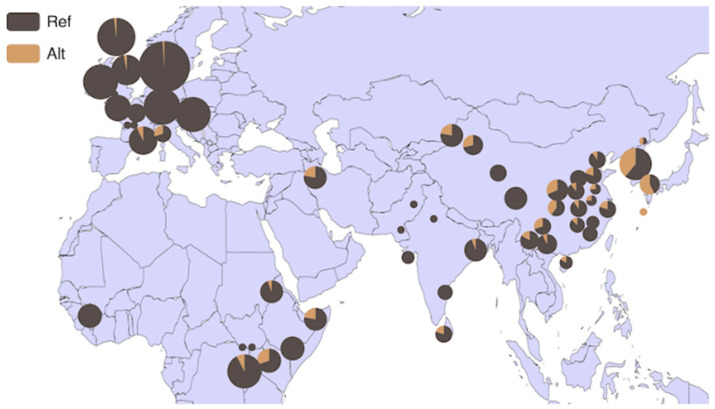
Allele frequency distribution of P4-D_22bp_ of HMGA2 in 54 cattle breeds worldwide. Note: REF: reference genotype; ALT: alternate genotype.

**Figure 6:**
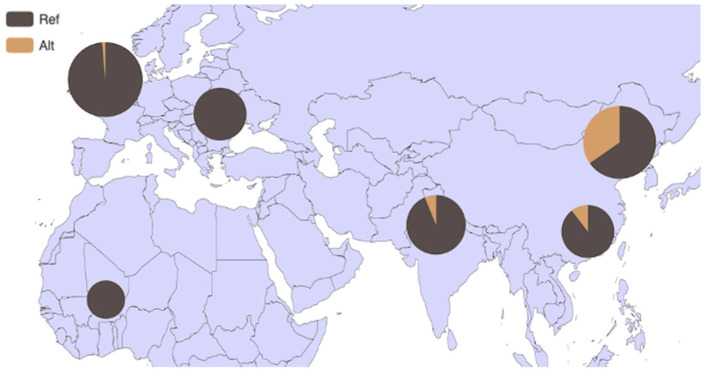
Allele frequency distribution of P4-D_22bp_ of *HMGA2* in six core cattle breeds worldwide. Note: REF: reference genotype; ALT: alternate genotype.

Based on the BGVD database (http://animal.omics.pro/code/index.php/BosVar, accessed on 3 July 2022), the distribution of the *HMGA2* gene in 54 bovine breeds worldwide is illustrated in Figure 5. This gene exhibits a high frequency in the Mishima (frequency = 0.571), Bashan (frequency = 0.400), and Hanwoo (frequency = 0.389) breeds of cattle. Subsequently, we focused on six core bovine breeds from the database, revealing a strong regional specificity. In comparison to Europe (frequency = 0.013), the gene demonstrates higher frequency in East Asian breeds (frequency = 0.347) (Figure 6).

## 4. Discussion

Ovaries play a pivotal role in the production of oocytes, a critical component in the reproductive cycle. Additionally, they contain corpus luteum and various components intricately linked to female reproductive endocrine regulation [26,27]. The primary roles of the ovaries involve generating developmentally competent oocytes capable of fertilization, leading to successful conception, and producing sex steroid hormones crucial for initiating and maintaining the estrous cycle as well as supporting pregnancy. The concept of an ovarian reserve refers to the inherent capability of these functions and is recognized as a critical determinant of fertility in mammalian females, including both humans and cattle. This reserve is a key factor in assessing reproductive potential and health in these species [28]. More importantly, the morphological characteristics of the ovary are closely related to the quantity of follicles, exerting an indirect influence on female animal fertility. Enhancing our knowledge of ovarian characteristics is crucial for advancing animal fertility, which is a pivotal factor in increasing the profitability of the animal breeding industry [29]. Scientists have explored the association between ovarian traits and polymorphic mutations. For example, studies have shown that the *SEPT7* gene is closely related to ovarian length [30], and other studies have demonstrated a connection between the *HSD17B3* gene and ovary morphological traits as well as mature follicle number [31]. The *HMGA2* gene is regarded as a candidate gene for improving female fertility, whereas its function still needs further verification. 

Up to now, most studies into the association between *HMGA2* and animal reproduction have predominantly focused on diseases, including PCOS and ovarian serous papillary carcinoma, as previously mentioned. At the same time, through the database of RGD (http://animal.omics.pro/code/index.php/RGD, accessed on 4 July 2022),we observed that the *HMGA2* gene is expressed in the embryonic gonadal ridge, which is related to ovarian traits (Figure S2). The results of our current study strongly suggest a significant relationship between mutations in the *HMGA2* gene and ovarian traits, signaling a potential association between this gene and ovarian characteristics. Furthermore, our results align with previous GWAS results that identified the *HMGA2* gene as a candidate gene for fertility. Specifically, as previous GWAS studies have shown, microRNA-33b-5p (MIR-33b-5p) is related to the pathogenesis in PCOS where *HMGA2* is also involved. MIR-33b-5p can inhibit glucose transporter 4 (GLUT4) production by targeting *HMGA2* [32]. In addition, the *HMGA2* gene is significantly involved in modulating the GLUT4 expression [32]. The *HMGA2* gene is significantly associated with GLUT4, a transporter responsible for glucose absorption in skeletal muscle and adipose tissue in insulin responsive tissues [32]. GLUT4 plays a key role in glucose metabolism in organisms. The connection between *HMGA2* and GLUT4, along with their influence on the ovary through microRNA-33b-5p, suggests that *HMGA2* may ultimately impact ovarian traits by affecting GLUT4.

Additionally, research has identified *HMGA2*, a protein typically present during fetal development, as being excessively expressed in ovarian cancer cases [33]. Furthermore, the prevalence of heightened *HMGA2* levels is observed in both initial and advanced stages of high-grade ovarian serous papillary carcinoma [34]. Moreover, *HMGA2* plays a significant role in regulating cell growth and differentiation, especially during the embryonic stage [35]. This may be another explanation for the close association between the *HMGA2* gene and fertility. An analysis of the frequency of the *HMGA2* gene in different breeds revealed regional specificity: it is more widely expressed in bovine breeds in Asia compared to European breeds. This phenomenon may be due to the different uses of bovine and the selection for desired traits. Simultaneously, through amino acid sequence alignment, we found that the *HMGA2* gene is highly conserved in cattle and humans (Figure S3). Previously, we discussed the possible relationship between the *HMGA2* gene and human reproductive PCOS, which suggests that *HMGA2* may affect ovarian traits in cattle. To ensure the accuracy and relevance of the analysis, we conducted a phased analysis of ovarian traits according to the estrus cycle, and our results indicate that this mutation site (P4-D_22bp_) in the *HMGA2* gene is related to ovarian traits during diestrus. However, previous studies have shown that *HMGA2* may be associated with PCOS, which can lead to the disruption of the estrous cycle. Meanwhile, due to the fact that the trait is controlled by multiple microgenes, our exploration was limited to examining the association between this locus and bovine ovarian traits. The specific nature of their association requires further dedicated study.

Moreover, although the mutation is situated in the fourth intron of the gene, numerous prior studies have highlighted that intronic mutations may be associated with traits. For instance, studies have shown that the introns of the *IGF2* gene may affect muscle growth in pigs [36]. Similarly, the mutation in the intron of the *DENDD1A* gene has been linked to bovine fertility [37], showcasing parallels with our study. In our investigation, this 22 bp short fragment deletion is located in the intron region, lacking a direct influence on protein sequence. Nevertheless, it may still be connected to specific transcription factors, influencing gene expression. In summary, our research establishes a relationship between the newly discovered indel in the *HMGA2* gene and various ovarian characteristics in cattle. This indel holds promise for effective utilization in marker-assisted selection (MAS) for bovine breeding purposes. However, to gain a deeper insight into how this indel influences bovine fertility, additional functional studies are required. Fertility constitutes a multifaceted characteristic influenced by a multitude of genes and genetic variations. Through the examination of specific indel loci and their associations with ovarian attributes, our objective is to offer insights into the utilization of molecular marker-assisted selection (MAS) for enhancing bovine female fertility. This advancement holds the potential to expedite progress within the cattle sector.

## 5. Conclusions

In conclusion, our research underscores the discovery of a novel indel locus within the *HMGA2* gene in Holstein cows. Notably, we elucidated its association with ovarian length and ovarian height during diestrus. These findings represent a distinctive contribution to the field of cattle breeding, offering unique insights and emphasizing the diverse applications of genetic methodologies in advancing the industry.

## Figures and Tables

**Table 1:** PCR primer sequences for bovine *HMGA2* amplification.

Loci	Rs Numbers	Primer Sequences (5′-3′)	Product Size (bp)	Tm (°C)	Region
P1-I_8-bp_	rs516271779	F: GGATTTGCGAGCAAGTCT	111/95	TD-PCR	5′UTR
		R: TACCCTCCTGGCAGATTG			
P2-D_12-bp_	rs797520182	F: CATTCTCCAGCCCTACCTCG	172	TD-PCR	5′UTR
		R: GTTGTCCCTGGGCTGAAGTG			
P3-I_23-bp_	rs797609300	F: CAACGGTTCTGTTGAGCAGC	120	TD-PCR	Intron
		R: CACCGGTTGGCTCCTAGTTT			
P4-D_22-bp_	rs133750033	F:AGAGCCTTTCTAGCAGTAGGTG	172/194	TD-PCR	Intron
		R: CAAGGTAAAGGGGGCTTTGG			

Note: P means pairs of primers and the numbers represent the position order of the detected potential polymorphic locus. D means deletion, and I means insertion.

**Table 2:** Estimation of polymorphism parameters of P4-D_22-bp_ of bovine *HMGA2* gene.

Sample Size	Genotypic Frequencies			Allelic Frequencies		HWE *p* Value	Population Parameter Estimates			
	II	ID	DD	I	D		Ho	He	Ne	PIC
634	0.180	0.340	0.480	0.350	0.650	*p* < 0.05	0.543	0.457	1.840	0.352

Note: HWE, Hardy–Weinberg equilibrium; Ho, homozygosity; He, heterozygosity; Ne, effective number of alleles; PIC, Polymorphism information content.

**Table 3:** Relationships between P4-D_22-bp_ polymorphisms of *HMGA2* and ovarian morphological traits during diestrus (Mean ± SE).

Sample Sizes	Traits (Units)	Observed Genotypes (Mean ± SE)			*p*-Values
		II (n)	ID (n)	DD (n)	
339	Ovarian length (mm)	42.65 ^A^ ± 0.60	40.68 ^B^ ± 1.11	37.67 ^AB^ ± 1.39	0.001
339	Ovarian width (mm)	26.59 ^a^ ± 0.50	25.38 ^b^ ± 0.65	25.03 ^ab^ ± 0.87	0.172
339	Ovarian height (mm)	24.70 ^B^ ± 0.58	25.31 ^AB^ ± 0.72	27.82 ^A^ ± 1.13	0.026
338	Ovarian weight (g)	11.91 ± 0.38	11.52 ± 0.55	10.80 ± 0.68	0.356
341	Number of corpus lutea	1.83 ^a^ ± 0.08	1.75 ^ab^ ± 0.11	1.57 ^b^ ± 0.13	0.288
340	Diameter of corpus luteum (mm)	10.45 ± 0.54	10.30 ± 0.77	10.59 ± 0.88	0.971
285	Number of mature follicles	0.43 ^B^ ± 0.05	0.44 ^A^ ± 0.08	0.50 ^A^ ± 0.11	0.827
98	Diameter of mature follicle (mm)	12.99 ^B^ ± 0.58	13.11 ^A^ ± 0.98	11.68 ^A^ ± 1.15	0.539

Note: Values with different letters (a, b/A, B) within the same row differ significantly at *p* < 0.05/*p* < 0.01.

## Data Availability

Data are contained within the article.

## Supplementary Materials

The following supporting information can be downloaded at: https://www.mdpi.com/article/10.3390/ani14050796/s1, Figure S1: Electrophoresis pattern of bovine *HMGA2* gene of P_1_-I_8_-_bp_. Table S1: Polymorphism parameters of P_1_-I_8_-_bp_ of bovine *HMGA2* gene. Figure S2: Expression of *HMGA2* gene in bovine. Note: FPKM: Fragment Per Kilobase of transcript, per Million mapped reads. Figure S3: Phylogenetic tree based on the amino acid sequences of *HMGA2* among different species. Note: Bootstrap method: 1000.

## References

[1] Berglund B. (2008). Genetic improvement of dairy cow reproductive performance. Reprod. Domest. Anim..

[2] Devoto L., Henríquez S., Kohen P., Strauss J.F. (2017). The significance of estradiolmetabolites in human corpus luteum physiology. Steroids.

[3] Lande R., Thompson R. (1990). Efficiency of marker-assisted selection in the improvement of quantitative traits. Genetics.

[4] Lin S., Zhang H., Hou Y., Liu L., Li W., Jiang J., Han B., Zhang S., Sun D. (2019). SNV discovery and functional candidate gene identification for milk composition based on whole genome resequencing of Holstein bulls with extremely high and low breeding values. PLoS ONE.

[5] Gershon E., Dekel N. (2020). Newly Identified Regulators of Ovarian Folliculogenesis and Ovulation. Int. J. Mol. Sci..

[6] Bai Y., Zhang F., Zhang H., Xu C., Wu L., Xia C. (2020). Follicular Fluid Metabolite Changes in Dairy Cows with Inactive Ovary Identified Using Untargeted Metabolomics. BioMed Res. Int..

[7] Aguiar T.S., Torrecilha R.B.P., Milanesi M., Utsunomiya A.T.H., Trigo B.B., Tijjani A., Musa H.H., Lopes F.L., Ajmone-Marsan P., Carvalheiro R. (2018). Association of Copy Number Variation at Intron 3 of HMGA2 With Navel Length in Bos indicus. Front. Genet..

[8] Lango Allen H., Estrada K., Lettre G., Berndt S.I., Weedon M.N., Rivadeneira F., Willer C.J., Jackson A.U., Vedantam S., Raychaudhuri S. (2010). Hundreds of variants clustered in genomic loci and biological pathways affect human height. Nature.

[9] Wang X., Wang J., Zhao J., Wang H., Chen J., Wu J. (2022). HMGA2 facilitates colorectal cancer progression via STAT3-mediated tumor-associated macrophage recruitment. Theranostics.

[10] Mansoori B., Mohammadi A., Ditzel H.J., Duijf P.H.G., Khaze V., Gjerstorff M.F., Baradaran B. (2021). HMGA2 as a Critical Regulator in Cancer Development. Genes.

[11] Boyko A.R., Quignon P., Li L., Schoenebeck J.J., Degenhardt J.D., Lohmueller K.E., Zhao K., Brisbin A., Parker H.G., von Holdt B.M. (2010). A simple genetic architecture underlies morphological variation in dogs. PLoS Biol..

[12] Makvandi-Nejad S., Hoffman G.E., Allen J.J., Chu E., Gu E., Chandler A.M., Loredo A.I., Bellone R.R., Mezey J.G., Brooks S.A. (2012). Four loci explain 83% of size variation in the horse. PLoS ONE.

[13] Chung J., Zhang X., Collins B., Sper R.B., Gleason K., Simpson S., Koh S., Sommer J., Flowers W.L., Petters R.M. (2018). High mobility group A2 (HMGA2) deficiency in pigs leads to dwarfism, abnormal fetal resource allocation, and cryptorchidism. Proc. Natl. Acad. Sci. USA.

[14] Liu M., Hummitzsch K., Hartanti M.D., Rosario R., Bastian N.A., Hatzirodos N., Bonner W.M., Irving-Rodgers H.F., Laven J.S.E., Anderson R.A. (2020). Analysis of expression of candidate genes for polycystic ovary syndrome in adult and fetal human and fetal bovine ovaries. Biol Reprod..

[15] Ferrero H. (2020). HMGA2 involvement in uterine leiomyomas development through angiogenesis activation. Fertil. Steril..

[16] Wei J.J. (2022). HMGA2: A Biomarker in Gynecologic Neoplasia. J. Clin. Transl. Pathol..

[17] Li M., Zhao H., Zhao S.G., Wei D.M., Zhao Y.R., Huang T., Muhammad T., Yan L., Gao F., Li L. (2019). The HMGA2-IMP2 Pathway Promotes Granulosa Cell Proliferation in Polycystic Ovary Syndrome. J. Clin. Endocrinol. Metab..

[18] Neupane M., Geary T.W., Kiser J.N., Burns G.W., Hansen P.J., Spencer T.E., Neibergs H.L. (2017). Loci and pathways associated with uterine capacity for pregnancy and fertility in beef cattle. PLoS ONE.

[19] Bachelot A. (2016). Polycystic ovarian syndrome: Clinical and biological diagnosis. Ann. Biol. Clin..

[20] Reynolds L.P., Redmer D.A. (1999). Growth and development of the corpus luteum. J. Reprod. Fertil. Suppl..

[21] Aljanabi S.M., Martinez I. (1997). Universal and rapid salt-extraction of high quality genomic DNA for PCR-based techniques. Nucleic Acids Res..

[22] Akhatayeva Z., Mao C., Jiang F.G., Pan C.Y., Lin C.J., Hao K.J., Lan T.X., Chen H., Zhang Q.F., Lan X.Y. (2020). Indel variants within the PRL and GHR genes associated with sheep litter size. Reprod. Domest. Anim..

[23] Hui Y., Zhang Y., Wang K., Pan C., Chen H., Qu L., Song X., Lan X. (2020). Goat DNMT3B: An indel mutation detection, association analysis with litter size and mRNA expression in gonads. Theriogenology.

[24] Li Z., Zhang Z., He Z., Tang W., Li T., Zeng Z., He L., Shi Y.Y. (2009). A partition-ligation-combination-subdivision EM algorithm for haplotype inference with multiallelic markers: Update of the SHEsis. Cell Res..

[25] Chen N., Fu W., Zhao J., Shen J., Chen Q., Zheng Z., Chen H., Sonstegard T.S., Lei C., Jiang Y. (2020). BGVD: An Integrated Database for Bovine Sequencing Variations and Selective Signatures. Genom. Proteom. Bioinform..

[26] Cao C., Zhou Q., Kang Y., Zhanerke A., Liu P., Bai Y., Li R., Jiang Y., Zhang Q., Lan X. (2024). A repertoire of single nucleotide polymorphisms (SNPs) of major fecundity BMPR1B gene among 75 sheep breeds worldwide. Theriogenology.

[27] Sharma R.K., Singh P., Setia A., Sharma A.K. (2020). Insecticides and ovarian functions. Environ. Mol. Mutagen..

[28] Nagai K., Yanagawa Y., Katagiri S., Nagano M. (2016). The relationship between antral follicle count in a bovine ovary and developmental competence of in vitro-grown oocytes derived from early antral follicles. Biomed. Res..

[29] Franchi F.F., Hernandes M.P., Coalho Ferreira A.L., Vieira de Lima V.A., de Oliveira Mendes L., Musa de Aquino A., Scarano W.R., César de Souza Castilho A. (2020). Fractal analysis and histomolecular phenotyping provides insights into extracellular matrix remodeling in the developing bovine fetal ovary. Biochem. Biophys. Res. Commun..

[30] Lan K., Shen C., Li J., Zhang S., Lan X., Pan C., Wang Y. (2023). A novel indel within the bovine SEPT7 gene is associated with ovary length. Anim. Biotechnol..

[31] Li J., Zhang S., Shen C., Niu Z., Yang H., Zhang K., Liu Z., Wang Y., Lan X. (2021). Indel mutations within the bovine HSD17B3 gene are significantly associated with ovary morphological traits and mature follicle number. J. Steroid Biochem. Mol. Biol..

[32] Yang Y., Jiang H., Xiao L., Yang X. (2018). MicroRNA-33b-5p is overexpressed and inhibits GLUT4 by targeting HMGA2 in polycystic ovarian syndrome: An in vivo and in vitro study. Oncol. Rep..

[33] Mahajan A., Liu Z., Gellert L., Zou X., Yang G., Lee P., Yang X., Wei J.J. (2010). HMGA2: A biomarker significantly overexpressed in high-grade ovarian serous carcinoma. Mod. Pathol..

[34] Wu J., Liu Z., Shao C., Gong Y., Hernando E., Lee P., Narita M., Muller W., Liu J., Wei J.J. (2011). HMGA2 overexpression-induced ovarian surface epithelial transformation is mediated through regulation of EMT genes. Cancer Res..

[35] Hock R., Witte F., Brocher J., Schütz M., Scheer U. (2006). Expression of HMGA2 variants during oogenesis and early embryogenesis of Xenopus laevis. Eur. J. Cell Biol..

[36] Van Laere A.S., Nguyen M., Braunschweig M., Nezer C., Collette C., Moreau L., Archibald A.L., Haley C.S., Buys N., Tally M. (2003). A regulatory mutation in IGF2 causes a major QTL effect on muscle growth in the pig. Nature.

[37] Zheng J., Deng T., Jiang E., Li J., Wijayanti D., Wang Y., Ding X., Lan X. (2021). Genetic variations of bovine PCOS-related DENND1A gene identified in GWAS significantly affect female reproductive traits. Gene.

