# Supplementary material for: Exploration of the Polymorphism Distribution of Bovine HMGA2 Gene in Worldwide Breeds and Its Associations with Ovarian Traits
Source: Animals (Basel). 2024 Mar 4;14(5):796. doi: 10.3390/ani14050796 (PMC10930529; doi:10.3390/ani14050796)
Supplement: Supplementary file 1 [file animals-14-00796-s001.zip › animals-2885739-supplementary.pdf]

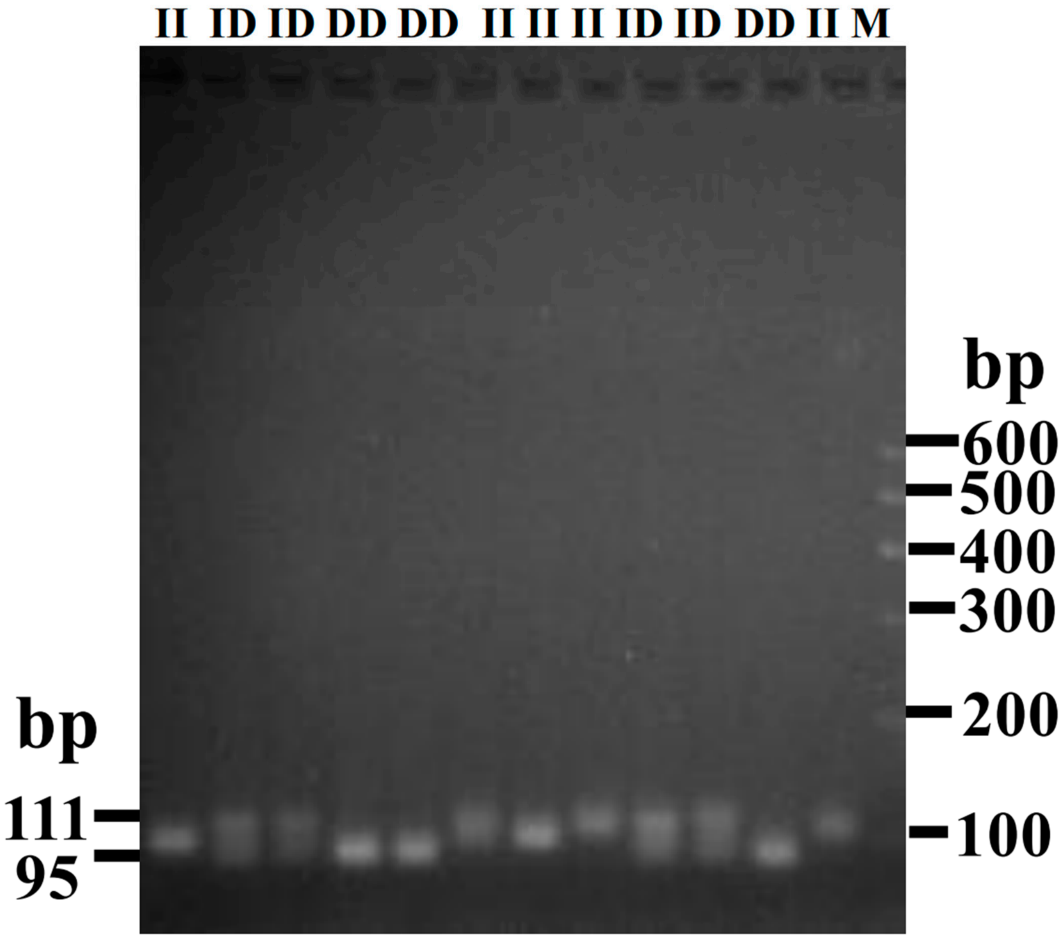

Figure S1. Electrophoresis pattern of bovine *HMGA2* gene of P1-$I_{8\text{-bp}}$

Table S1. Polymorphism parameters of P1-$I_{8\text{-bp}}$ of bovine *HMGA2* gene.

Size*	Genotypic frequencies			Allelic frequencies		HWE *P* value	Population parameters			
	II	ID	DD	I	D		Ho	He	Ne	PIC
130	0.22	0.26	0.52	0.35	0.65	$P<0.05$	0.52	0.48	1.840	0.35

Note: * refers to the number of individual bovine analyzed. ***HWE***, Hardy-Weinberg equilibrium; ***Ho*****,** homozygosity; ***He*****,** heterozygosity; ***Ne*****,** effective number of alleles; ***PIC*****,** Polymorphism information content. **Sizes**: the number of individual cattle analyzed.

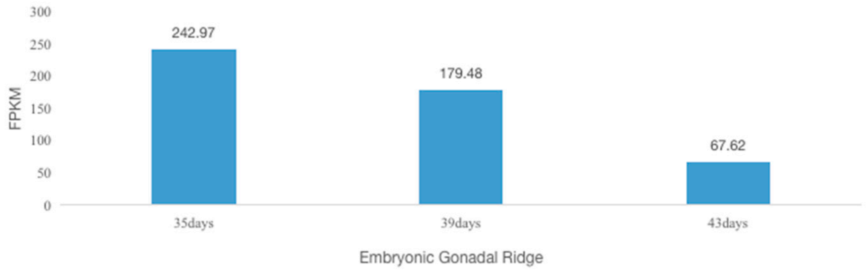

Figure S2. Expression of *HMGA2* gene in bovine. Note: FPKM: Fragment Per Kilobase of transcript, per Million mapped reads

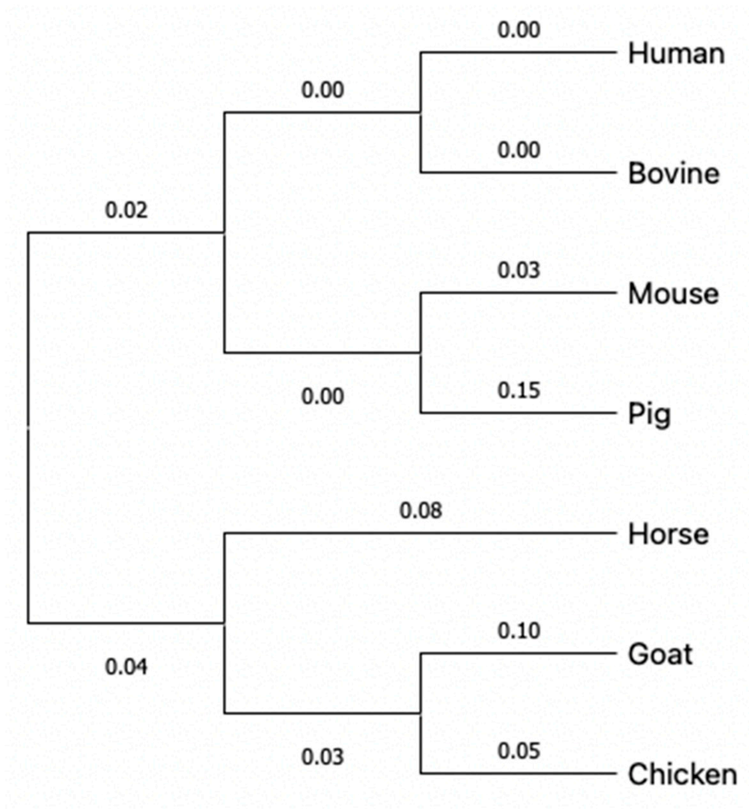

Figure S3. Phylogenetic tree based on the amino acid sequences of HMGA2 among different species. Note: Bootstrap method: 1000
